# The Influence of CoO/P_2_O_5_ Substitutions on the Structural, Mechanical, and Radiation Shielding of Boro-Phosphate Glasses

**DOI:** 10.3390/ma14216632

**Published:** 2021-11-03

**Authors:** Ahmed M. A. Mostafa, Mohamed A. M. Uosif, Ziyad A. Alrowaili, Reda Elsaman, Ahmed A. Showahy, Yasser B. Saddeek, Shams A. M. Issa, Antoaneta Ene, Hesham M. H. Zakaly

**Affiliations:** 1 Physics Department, College of Science, Jouf University, Sakaka P.O. Box 2014, Saudi Arabia; mauosif@ju.edu.sa (M.A.M.U.); Zalrowaili@ju.edu.sa (Z.A.A.); 2 Physics Department, Faculty of Science, Al-Azhar University, Assiut 71524, Egypt; RedaElsayed536.el@azhar.edu.eg (R.E.); dra.a.showahy@azhar.edu.eg (A.A.S.); y.mohamed@mu.edu.sa (Y.B.S.); sh_issa@ut.edu.sa (S.A.M.I.); 3 Physics Department, College of Science in Zulfi, Majmaah University, Al Majma’ah 11952, Saudi Arabia; 4 Physics Department, Faculty of Science, University of Tabuk, Tabuk 47512, Saudi Arabia; 5 INPOLDE Research Center, Department of Chemistry, Physics and Environment, Faculty of Sciences and Environment, Dunarea de Jos University of Galati, 47 Domneasca Street, 800008 Galati, Romania; 6 Institute of Physics and Technology, Ural Federal University, 620002 Ekaterinburg, Russia

**Keywords:** polymerization of the boro-phosphate glass network, CoO/P_2_O_5_, radiation protection

## Abstract

A new glass system (50−x)P_2_O_5_–20B_2_O_3_–5Al_2_O_3_–25Na_2_O–xCoO was manufactured using a standard melt quenching procedure, where 1≤ x ≤ 12 mol%. The characteristics of boro-phosphate-glasses containing CoO have been studied. The effect of CoO on the radiation-protective properties of glasses was established. The density of the prepared glasses as a function of CoO increased. XRD was used to check the vitreous structure of samples. Fourier-transform infrared (FTIR) spectroscopy was used to study the structure of each sample. FTIR demonstrated that connections grew as CoO/P_2_O_5_ levels increased, and the FTIR spectra shifted to higher wavenumbers. The increment of CoO converts non-bridging oxygens associated with phosphate structural units into bridging oxygens. This process increases the concentration of BO_4_ structural units and creates new, strong and stable bonds B–O–P, i.e., there is polymerization of the boro-phosphate glass network. With an increase in the ratio of CoO/P_2_O_5_ in the produced samples, ultrasonic velocities and elastic moduli were observed to increase. The coefficients of linear and mass attenuation, the transmittance of photons in relation to the photon energy, the efficiency of radiation protection in relation to the photon energy, and the thickness of the absorber were modeled using these two methods (experimental and theoretical). From the obtained values, it can be concluded that the 12Co sample containing 12 mol% will play the most influential role in radiation protection. An increase in the content of cobalt-I oxide led to a significant increase in the linear and mass attenuation coefficient values, which directly contributes to the development of the radiation-protective properties of glass.

## 1. Introduction

The development of materials for protection against nuclear radiation has become necessary due to the broader use of radioactive materials in medicine, agriculture, and industry. Compounds can be used to protect against nuclear radiation if these compounds have sufficient ability to absorb this radiation to a safe level [1]. Recently, glass materials have been one of the potential options for contrasting with concrete, as they serve the dual purpose of providing visibility while simultaneously absorbing radiation such as gamma rays and neutrons. Moreover, knowledge of interacting factors such as mass attenuation coefficient, gamma interaction cross-section, effective atomic number (Z_eff_), and electron density (N_el_) is vital in connection with the rapidly growing use of radioactive isotopes in agriculture, medicine, and manufacturing [2].

The up-to-date expertise of the alkali boro-phosphate glass industry has enhanced the lives of people worldwide. Good chemical durability characterizes these glasses, along with their mechanical stability, low refractive index, and thermal parameters either in preparation or characteristic temperatures (e.g., softening and glass transition temperatures). Moreover, multi-component boro-phosphate glasses with the Al_2_O_3_, Na_2_O, or transition metal oxides make these glasses good candidates for optical tools, rechargeable batteries, super-capacitors, photochromic windows, bioactive materials, and the immobilization of radioactive waste [3,4,5,6,7].

The addition of CoO with different valence states and at different concentrations to the phosphate glasses enhances their properties [8]. Co^2+^ ion is characterized by the presence of 3d^7^ electronic configuration and can be involved in different and important applications due to its possible transformation from tetrahedral to octahedral states and vice versa [9,10]. Such a transformation is useful in the optical and thermal studies of phosphate glasses involving CoO [11]. Based on the valence state, CoO can take up substitutional or interstitial positions in the phosphate glass network. The entrance of Co^2+^ ions in substitutional positions may grow more cross-linkages in the glass network [12]. The addition of B_2_O_3_ to ultra-phosphate-based glasses sets the formation of BPO_4_ groups because the BO_3_/BO_4_ ratio has a critical influence on the structure. The structural complexity of these glasses increases with the addition of several oxides in their network. This complexity arises from the chemical interaction between oxygens from these oxides and the phosphate or the borate structural units. The existence of the third constituent in boro-phosphate-based glasses initiated noteworthy variations in both their structural network and their physical properties. The expected changes are due to the cross-linking of the structural units or the bonding arrangements between borate and phosphate structural units [13,14].

Moreover, the elastic features of a glass network are correlated to any changes, in the structural units, of the types of bonds in such a network. Such elastic features are necessary in the estimation of the potential of these glasses for an unlimited range of uses, for example, radiation shielding [15,16,17]. The current work was interested in the variations in CoO content that take place within the glass system (50−x)P_2_O_5_–20B_2_O_3_–5Al_2_O_3_–25Na_2_O–xCoO (x = 1, 2, 4, 8 and 12 mol%) and their effects on the structure of these glasses. Moreover, the elastic features and the radiation shielding parameters were also considered.

## 2. Materials and Methods

Glass samples of a new chemical formula of (50−x)P_2_O_5_–20B_2_O_3_–5Al_2_O_3_–25Na_2_O–xCoO, where x = 1, 2, 4, 8 and 12 (mol%), were obtained by the conventional melt quenching method. The nominal compositions are shown in Table 1. The prepared samples had a thickness ranging from 0.35 to 0.65 cm.

The materials that initiated the reaction were the reagent class (NH_4_)_2_HPO_4_, Na_2_CO_3_, Al_2_O_3_, CoO, and H_3_BO_3_ (Sigma Aldrich, Vienna, Austria). Powdered oxides were melted at a temperature of 1050–1150 °C in a porcelain vessel in an electric oven (Lilienthal, Nabertherm, Germany) for 50 min. Next, the molten samples were poured into the preheated mold at 350 °C. Then, the prepared samples were ground and polished to measure the parameters of ultrasonic and radiation shielding. A Philips PW/1710 X-ray diffractometer (Philips, Cairo, Egypt) with Ni-filtered Cu-Kα radiation (λ = 1.54 Å) was used, and the Fourier transform infrared (FTIR) spectra were detected using a JASCO spectrophotometer (JASCO, Amsterdam, The Netherlands) in the range of 2000–400 cm^−1^. The thermal analysis of the obtained glasses as a function of CoO was determined by the standard Shimadzu differential scanning calorimeter (DSC 50) (Shimadzu, Kyoto, Japan). The 15 mg powdered glass sample was placed in a platinum crucible and examined up to 500 °C in Argon medium with a 20 K/min heating rate. Powdered alumina was used as reference material. The accuracy in the measurements of Tg was ±2 K.

**Figure 1:**
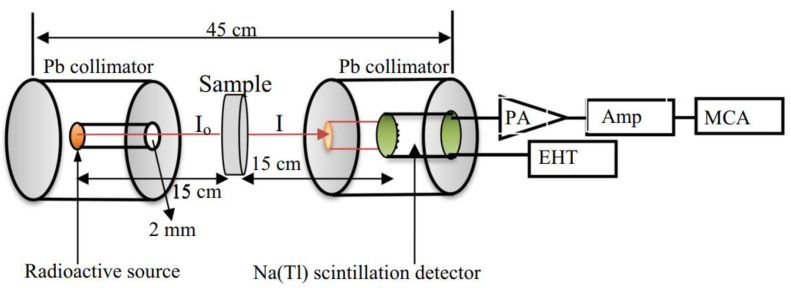
Experimental setup of gamma shielding measurements.

The linear attenuation coefficient (μ) of the fabricated glasses was measured using the NaI (Tl) spectroscopy system (Genie-2000 G2kBatch (S-6600), CANBERRA, Zellik, Belgium) shown in Figure 1. The gamma-ray shielding features were measured via 356, 662, 1173, and 1332 keV gamma emitted from Ba-133, Cs-137, and Co-60 (5 μCi) sources. The thickness of the Al window in the used detector was 0.05 cm and had a resolution of 7.5% at 0.662 meV energy emitted from Cs-137. All glasses were measured for about 4 h, repeated three times. 

## 3. Results and Discussions

**Figure 2:**
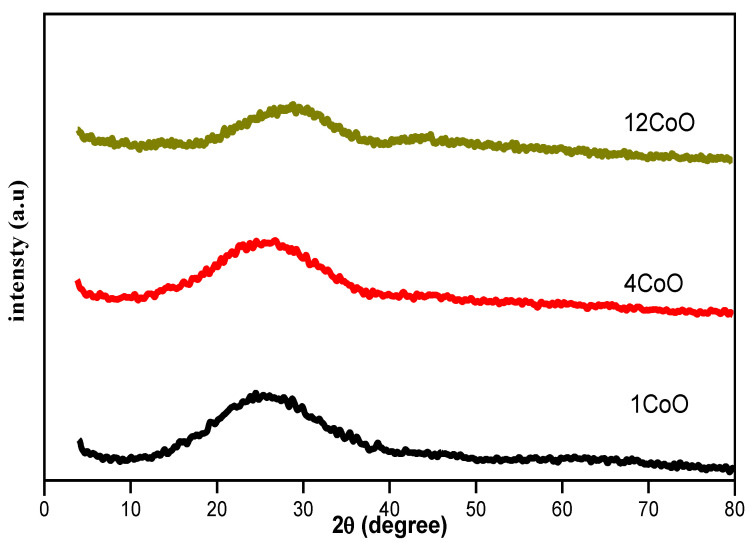
XRD patterns of the glasses (50−x)P_2_O_5_–20B_2_O_3_–5Al_2_O_3_–25Na_2_O–xCoO, (x = 1, 2, 4, 8 and 12 mol%).

XRD is often used to verify the glassy structure of samples. For example, as shown in Figure 2, the glass sample patterns presented an amorphous structure when no peaks were observed.

### 3.1. FTIR Analysis

**Figure 3:**
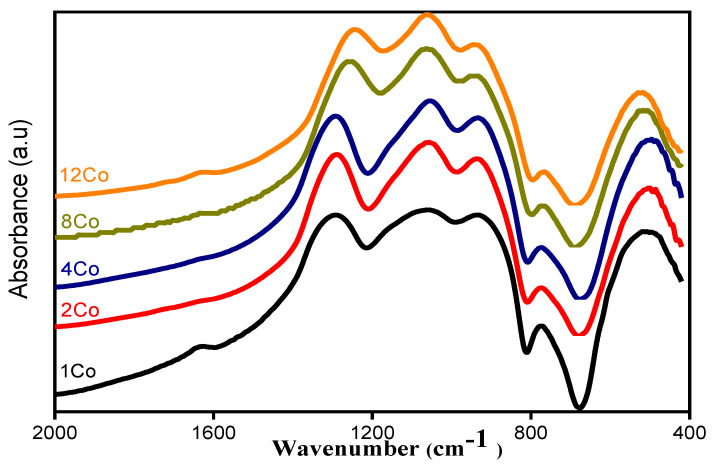
FTIR spectra of the glasses (50−x)P_2_O_5_–20B_2_O_3_–5Al_2_O_3_–25Na_2_O–xCoO, (x = 1, 2, 4, 8 and 12 mol%).

Figure 3 presents the FTIR spectra of the explored glasses. The spectra presented broad bands that were the result of overlapping between multiple bands. Each band was specified for a specific vibrational group. These broad bands should be deconvoluted into Gaussian profiles to present their original bands.

**Figure 4:**
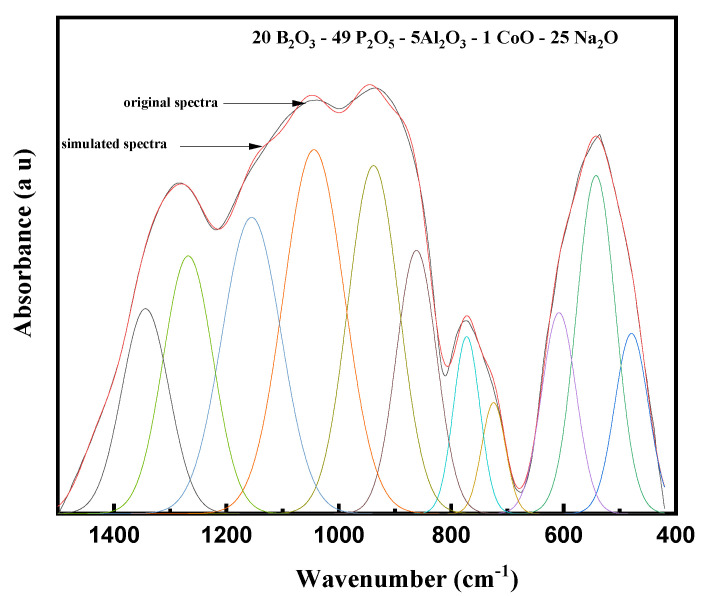
Curve fitting of FTIR spectra of the glasses 49P_2_O_5_–20B_2_O_3_–5Al_2_O_3_–25Na_2_O–1CoO.

**Figure 5:**
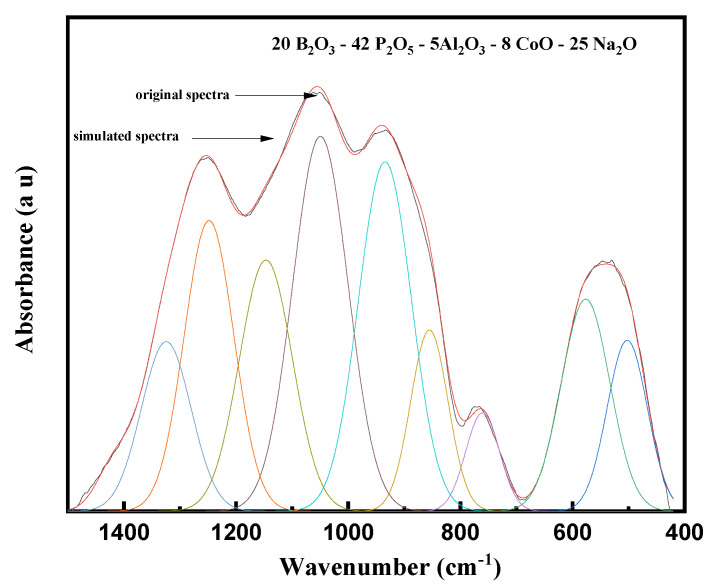
Curve fitting of FTIR spectra of the glasses 42P_2_O_5_–20B_2_O_3_–5Al_2_O_3_–25Na_2_O–8CoO.

Figure 4 and Figure 5 show the deconvolution spectra (red curves) of two glass samples, namely 1 Co and 8 Co, respectively. Based on the listed deconvolution parameters in Table 2, the band at 479 cm^−1^ is attributed to the bending vibrations of O–P–O in (PO^2−^)_n_ chain groups [18]. The band at 542 cm^−1^ is attributed to the vibrations of CoO_6_ units of Co^III^-O bonds [19,20]. The bands at 608 and 725 cm^−1^ are attributed to the fundamental frequency of (PO_4_)^−3^ and the harmonics of P=O bending vibrations [21,22]. The band around 772 cm^−1^ is attributed to bending vibrations of B–O–B bonds in BO_3_ groups [20].

Moreover, due to the presence of two glass formers, such as B_2_O_3_ and P_2_O_5_, in the network of the explored glasses, the FTIR spectra in regions 1200–800 cm^−1^ and 1500–1200 cm^−1^ had overlapping bands arising from the two oxides. Therefore, the bands appearing in the region 862–1155 cm^–1^ were due to the vibrations of the B–O bond stretching of BO_4_ structural units, which were overlapped with phosphate bands [23]. As mentioned before, the characteristic bands of phosphate in this region, i.e., at ~862, ~938, and ~1044 cm^−1^, were assigned to sy-stretching vibration of bridging oxygens in P–O–P bonds, sy-stretching vibrations of PO_4_, and to as-stretching of (PO3)^2−^ groups, respectively [24]. In the second region, i.e., the bands appearing in the region 1150–1450 cm^–1^ were attributed to the B–O bond stretching of trigonal BO_3_ units [25]. Therefore, the characteristic bands of phosphate in this region, i.e., at ~1155, ~1268, and ~1344 cm^−1,^ were assigned to the vibrations of PO_2_ sy-stretching mode, and as-P=O stretching vibrations to the vibrations of BO_3_/P=O groups [26,27].

The addition of CoO with different contents causes considerable variations in the relative intensity and position of these bands. The characteristic phosphate bands in the region 862–1155 cm^–1^ were shifted towards higher wavenumbers, while those in the region 1150–1450 cm^–1^ were shifted to lower wavenumbers. Furthermore, the intensities of these bands increased with the increase in CoO content. These observations confirm that in a glass system with B_2_O_3_ and P_2_O_5_, there is an opportunity for cross-linking BO_4_ and PO_4_ structural units. Thus, the increment in CoO converts non-bridging oxygens associated with phosphate structural units into bridging oxygens. This process increases the concentration of BO_4_ structural units and creates new, strong and stable bonds B–O–P, i.e., there is polymerization of the boro-phosphate glass network [20].

### 3.2. Analysis of Elastic Characteristics

**Figure 6:**
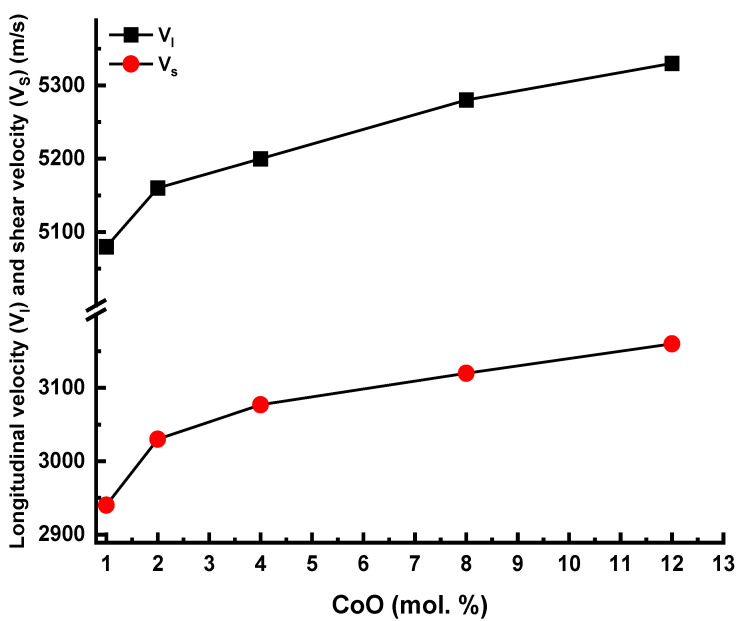
Longitudinal velocity (V_l_) and shear velocity (Vs) against glass composition.

Table 3 tabulates the values of the elastic characteristics. It is noted in Table 3 that the increment of CoO increases the measured values of the density and the ultrasonic velocities. The increase in the density with the increment of CoO concentrations is ascribed to the large changes in the density between CoO and P_2_O_5_ and is due to the polymerization of the boro-phosphate network, as discussed in the FTIR section. The polymerization created bridging oxygens and decreased the interatomic spacing, which increased the density in turn. As plotted in Figure 6, the increase in ultrasonic velocities can be attributed to the created high-strength B–O–P. Moreover, the movement of the FTIR bands to higher wavenumbers linked with the formation of bridging oxygens will compact the explore network and thus increase the rigidity of the boro-phosphate glass network. This evidence explains the increment of the ultrasonic velocities and Debye temperature, as tabulated in Table 3.

**Figure 7:**
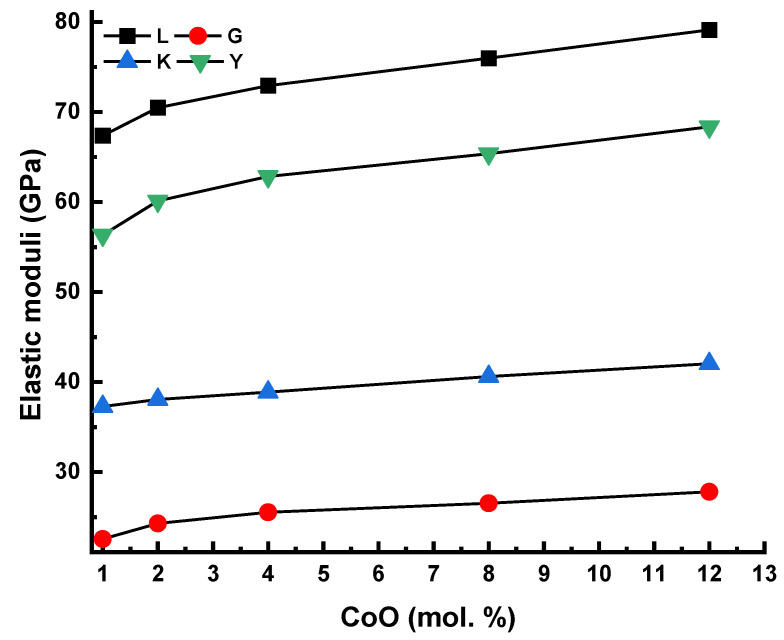
Longitudinal modulus (L), shear modulus (G), bulk modulus (K), and Young’s modulus as a function of the glass composition.

Figure 7 shows the increment of the elastic moduli as a function of CoO. Such an increment may be attributed to an increment in cross-link density and the number of bonds in the network. An additional reason for the increase in elastic properties is the shrinkage of the environment around Co^2+^ due to the enhanced field strength of structural units of linked BO_4_ and PO_4_ structural units.

### 3.3. Thermal Properties

**Figure 8:**
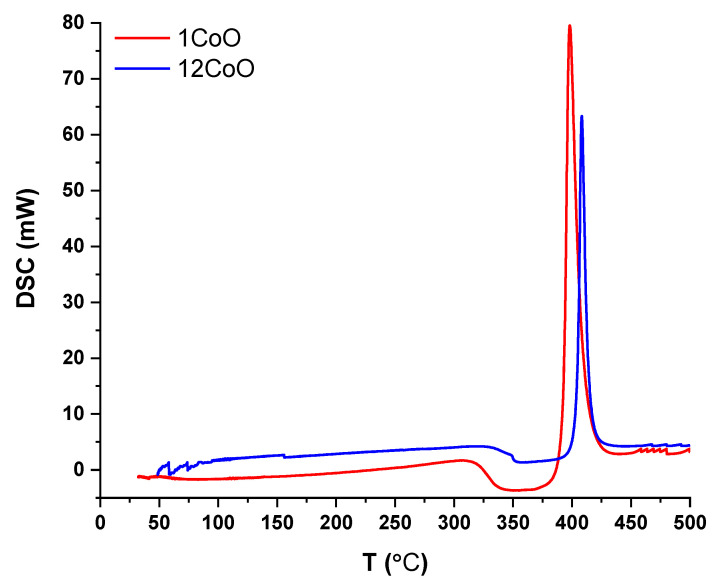
DSC curves for the explored glasses with various CoO concentrations.

DSC curves for the explored glasses with various CoO concentrations were obtained. Figure 8 shows the DSC traces of two samples, for example, 1CoO and 12CoO at a 20 K/min heating rate. The DSC curves revealed a single endothermic peak related to the glass transition temperature (Tg) and an exothermic peak attributed to the full crystallization temperature (Tc). The singular peak of Tg reveals good homogeneity of the prepared glasses. The Tg dependence on the CoO content, as shown in Figure 8, shows that the increase in CoO content causes an increase in Tg values. The increase in Tg could be related to the created bridging oxygens, the increment of cross-links between BO4 and PO4 structural units, and the polymerization of the boro-phosphate glass network [28,29,30].

### 3.4. Radiation Shielding Parameters

**Figure 9:**
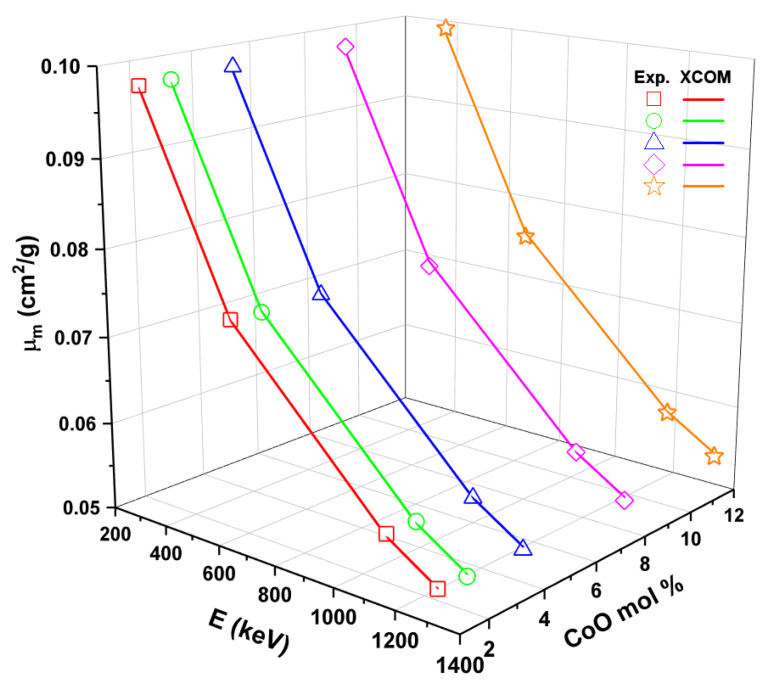
Experimental and theoretical mass attenuation coefficient (µ_m_) against glass composition.

In this work, five samples of glasses (1Co, 2Co, 4Co, 8Co, and 12Co) were studied in terms of their ability to protect against radiation. Figure 9 shows the experimental and theoretical values of the mass attenuation coefficient (μ_m_), varying with respect to the mol% of CoO in all samples. This figure shows that the µ_m_ values decrease with increasing energy. Thus, for example, as the CoO content increases from 1% to 12% (mol) in the glass samples, the bulk density of the glasses increases from 2.611 to 2.785 g/cm^3^ (see Table 1). At 356 keV, 0.09868, 0.09874, 0.09894, 0.09899 and 0.09910 (cm^2^/g) are the experimental μ_m_ values for 1Co, 2Co, 4Co, 8Co, and 12Co samples, respectively. The highest μ_m_ value was observed for the 12Co sample, with the highest CoO concentration in its chemical structure. This underscores the impact of increasing CoO concentration on gamma-ray shielding features.

**Figure 10:**
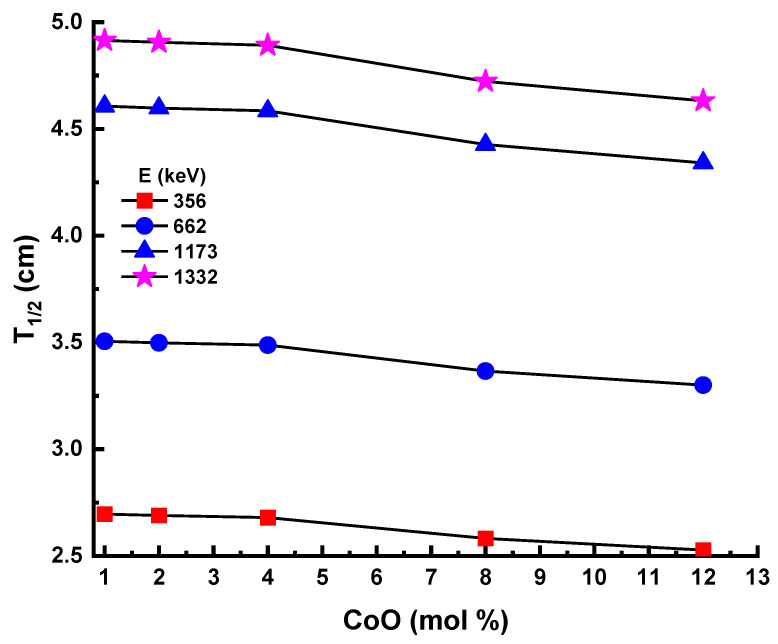
Experimental half-value layer (T_1/2_) for glass samples.

**Figure 11:**
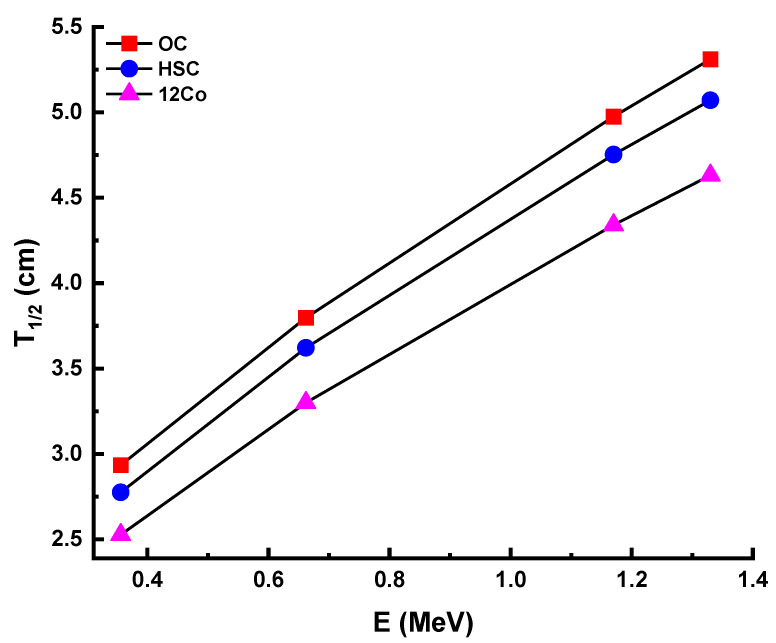
Comparison of experimental half-value layer (T_1/2_) value of the 12Co sample with both ordinary concrete (OC) and hematite-serpentine concrete (HSC) [31].

Figure 10 displays the behavior of the half-value layer values (T_1/2_) for the five test samples in the present study. This shows that the lowest T_1/2_ values were observed at 356 keV, while the highest T_1/2_ values were at 1332 keV. This could be described by the penetration potential of gamma rays, depending on their energy, which means that the low energy of gamma rays requires limiting the minimum thickness of the material. Moreover, the 12Co sample showed the lowest T_1/2_ values among the studied samples. These results confirm the excellent shielding performance of the 12Co sample among the prepared samples. As shown in Figure 11, the T_1/2_ values of the 12Co sample were lower than the T1/2 values of both ordinary concrete (OC) and hematite-serpentine concrete (HSC) [31].

**Figure 12:**
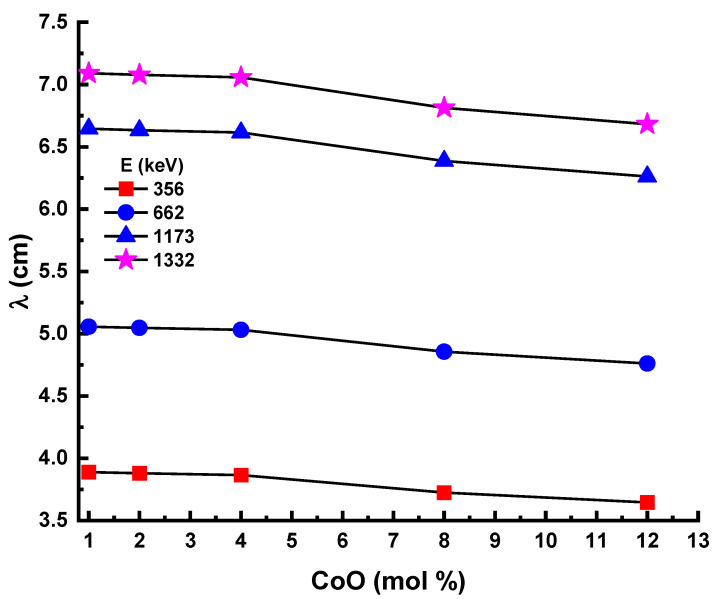
Experimental mean free path (λ) for glass samples.

Figure 12 shows the change in the experimental mean values of the free path (λ) depending on the photon energy for all samples. As with the trend for the T_1/2_ values, the lowest λ values were recorded for the 12Co sample.

**Figure 13:**
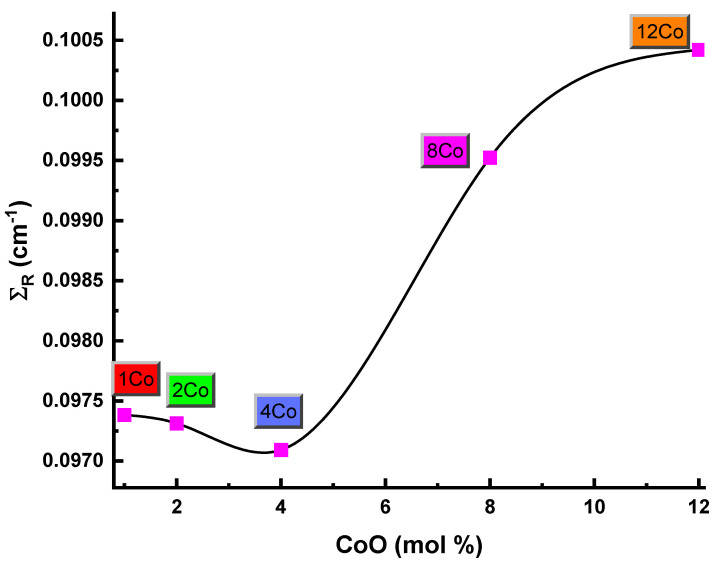
Removal cross sections for fast neutrons-Σ_R_ values of samples labeled as 1Co, 2Co, 4Co, 8Co, and 12Co for glass samples.

Figure 13 presents the removal cross sections for fast neutrons Σ_R_ values of samples labeled as 1Co, 2Co, 4Co, 8Co, and 12Co. It can be noted from Figure 13 that the concentration of Co was maximum in the high-density 12Co glass sample, and the extent of the group was proportional. That is to suggest, 12Co is more efficient in catching fast neutrons between glass samples.

## 4. Conclusions

The effect of the CoO/P_2_O_5_ ratio on the radiological, structural, and mechanical properties of the boro-phosphate P_2_O_5_–B_2_O_3_–Al_2_O_3_–Na_2_O_3_–CoO glass system was studied. As the CoO content increased from 1% to 12% (mol) in the glass samples, the bulk densities of glasses increased from 2.611 to 2.785 g/cm^3^. The analysis of FTIR spectroscopy confirmed that in a glass system with B_2_O_3_ and P_2_O_5_, there is an opportunity for cross-linking BO_4_ and PO_4_ structural units, and the increment of CoO converts non-bridging oxygens associated with phosphate structural units into bridging oxygens. This process increases the concentration of BO_4_ structural units and creates new, strengthened and stable bonds B–O–P, i.e., there is polymerization of the boro-phosphate glass network. Linear and mass attenuation coefficients, photon transmittance against photon energy, radiation protection efficiency against photon energy, and absorber thickness were simulated with the help of these two methods (experimental and simulation). From the obtained values, it can be concluded that the 12Co sample containing 12 mol% would have the most efficient role in radiation shielding. The cobalt-I-oxide increase resulted in a significant increase in linear and mass attenuation coefficient values, which contribute directly to the development of the radiation shielding properties of the glass. The study showed the positive effect of increasing cobalt-I-oxide on the mechanical and radiation shield properties. Furthermore, by increasing the CoO/P_2_O_5_ ratio at the expense of replacing phosphate pentoxide, the mechanical properties of the glass system also improved with the system’s ability to block and absorb gamma rays and X-rays. Thus, this study is a positive step to evaluate the glass systems used to protect against the dangers of ionizing radiation.

## Figures and Tables

**Table 1:** Chemical composition (mol.%) and density (*ρ*) of glasses.

Sample Code	B_2_O_3_	P_2_O_5_	Al_2_O_3_	CoO	Na_2_O	*ρ* (g/cm^3^)
1Co	20	49	5	1	25	2.611
2Co	20	48	5	2	25	2.617
4Co	20	46	5	4	25	2.627
8Co	20	42	5	8	25	2.726
12Co	20	38	5	12	25	2.785

**Table 2:** Deconvolution parameters of the glasses and their assignments. C is the center of the band, and A is the relative area (%) of the band.

**1Co**	**C**	479	542	608	725	772	862	938	1044	1155	1268	1344
	**A**	4.8	10.4	5.49	2.09	3.7	8.4	14	17.5	13.9	12.1	7.66
**2Co**	**C**	487	550	604	735	767	865	940	1049	1153	1286	1376
	**A**	6.9	8.81	3.58	1.05	2.8	8.2	15.8	18.5	13.7	15.8	4.85
**4Co**	**C**	474	540	597	724	768	859	933	1049	1157	1287	1383
	**A**	6.7	10	5.85	1.95	3.52	8.54	15.6	17.9	12.2	13.9	3.79
**8Co**	**C**	502	577	-	-	760	855	934	1050	1147	1248	1324
	**A**	6.59	9.9	-	-	3.2	6.51	17.8	19.9	12.8	15.2	8.11
**12Co**	**C**	511	591	-	-	753	852	935	1051	1137	1253	1388
	**A**	8.55	7.65	-	-	3.04	8.93	17	19.2	15.3	17.3	2.98

**Table 3:** Density, longitudinal velocity (V_l_), shear velocity (V_S_), longitudinal modulus (L), shear modulus (G), bulk modulus (K), Young’s modulus, Poisson’s ratio, Debye temperature, and Tg of the glasses.

Code	*ρ* (g/cm^3^)	V_l_ (m/s)	V_s_ (m/s)	L (GPa)	G (GPa)	σ	K (GPa)	Y (GPa)	Θ	Tg
1Co	2.611	5080	2940	67.38	22.57	0.25	37.29	56.34	422	314
2Co	2.647	5160	3030	70.48	24.30	0.24	38.08	60.12	436	319
4Co	2.697	5200	3077	72.93	25.54	0.23	38.88	62.85	444	325
8Co	2.726	5280	3120	75.99	26.54	0.23	40.62	65.37	451	331
12Co	2.785	5330	3160	79.12	27.81	0.23	42.039	68.36	457	338

## Data Availability

Data are contained within the article.
